# Mechanisms of Polymer–Antigen Binding and Hydrolysis Inhibition: Molecular Dynamics Simulations and Experimental Measurements

**DOI:** 10.3390/polym18070781

**Published:** 2026-03-24

**Authors:** Ziyang Hu, Kai Yue, Weishen Zhong, Genpei Zhang

**Affiliations:** 1 School of Energy and Environmental Engineering, University of Science and Technology Beijing, Beijing 100083, China; m202321718@xs.ustb.edu.cn (Z.H.); d202110075@xs.ustb.edu.cn (W.Z.); d202310087@xs.ustb.edu.cn (G.Z.); 2 Shunde Graduate School, University of Science and Technology Beijing, Foshan 528399, China

**Keywords:** polymers, MD simulation, polymer–protein interaction, hydrolysis inhibition

## Abstract

In situ cancer vaccines activate antitumor immune responses by locally capturing and presenting tumor-derived antigens, in which polymers play a key role as antigen-capturing materials. However, the influence of polymer composition and degree of polymerization (DP) on antigen capture efficiency and protection mechanisms remains insufficiently understood. In this study, the tumor-specific antigen MAGE-A3, highly expressed in esophageal squamous cell carcinoma (ESCC), was employed to investigate antigen capture and stabilization by five representative polymers—chitosan, polyethyleneimine (PEI), alginate, polycaprolactone (PCL), and poly (lactic-co-glycolic acid) (PLGA)—with different DPs, using molecular dynamics simulations and in vitro experiments. All-atom simulations revealed that hydrophobic interactions dominate polymer–antigen binding, while electrostatic interactions from cationic polymers synergistically enhance binding affinity and capture efficiency. Binding free energy analysis showed that van der Waals and electrostatic contributions stabilize the complexes, whereas polar solvation partially counteracts these effects. Experimentally, low-DP chitosan exhibited the highest antigen-capture efficiency (38.9%), attributed to its small molecular size, enabling multipoint binding across the antigen surface. In contrast, high-DP polymers generated pronounced steric hindrance that suppressed antigen–enzyme interactions and inhibited hydrolysis. These findings clarify how polymer composition and chain length jointly regulate antigen capture and protection, providing mechanistic guidance for the rational design of polymer-based in situ cancer vaccines.

## 1. Introduction

In recent years, cancer vaccines have emerged as a promising immunotherapeutic agent demonstrating significant potential in treating various advanced solid tumors [1,2,3,4]. Compared with other immunotherapies, cancer vaccines can induce more specific immune responses against tumor antigens. They contribute to the elimination of primary tumors and the suppression of distant metastases [5,6]. Conventional cancer vaccines typically rely on ex vivo antigen loading, a process that is operationally complex, costly, and often targets a single antigen [7,8], limiting their application. In contrast, in situ antigen-capturing vaccines activate the immune system directly within the tumor microenvironment by capturing and presenting antigens, thereby enhancing antitumor efficacy [9,10,11]. Polymers are extensively utilized in the development of in situ antigen-capture vaccines due to their tunable composition and size, which can be optimized to enhance interactions with antigens [12]. Therefore, elucidating the influence of polymer composition and polymerization degree on antigen capture is crucial for designing and optimizing novel in situ vaccines.

In the field of in situ antigen-capture vaccines, extensive studies have been conducted around the development of functional polymeric carriers, leading to substantial progress in both in vitro and animal experiments. Research indicates that using polymers as carriers or modifying nanoparticle surfaces can effectively achieve antigen capture in situ [13]. Among these biomaterials, FDA-approved poly (lactic-co-glycolic acid) (PLGA) microspheres, chitosan nanoparticles, and polyethylene glycol–polylactic acid (PEG-PLA) micelles have demonstrated excellent antigen-capturing performance and biocompatibility for the development of in situ antigen-capture vaccines and have also been evaluated in clinical studies [12]. A recent review summarized the progress of in situ vaccine delivery technologies, emphasizing the integrated “capture–delivery–activation” design of polymeric, lipid, and hydrogel systems and the influence of key parameters such as particle size, surface charge, and stimulus responsiveness on immune responses [14]. For example, lipid–polymer hybrid nanoparticles such as DOTAP-hNPs can capture negatively charged tumor antigens through electrostatic interactions and facilitate their delivery to antigen-presenting cells [15]. In addition, photothermal components such as AuNRs [16] and ICG [17] have been incorporated into polymer systems to promote tumor-antigen release and subsequent capture under stimulation conditions. These studies support the potential of polymers in antigen delivery and immune activation. Although these studies demonstrate the promise of polymer-based antigen-capture systems, the optimization of capture efficiency and antigen-binding stability remains insufficiently investigated.

In addition to their extensive application in antigen capture, polymers serve as protective agents that significantly reduce the risk of antigen proteolytic degradation in vivo [18,19]. When polymer chains bind to antigen surfaces, they can form a protective interfacial layer that hinders protease access to cleavage sites [20]. Enhancing these interactions, particularly electrostatic interactions between polymers and antigens, represents an effective strategy for improving antigen protection. Electrostatic interactions between positively charged polymers and negatively charged antigen proteins on the surface promote tight adsorption of the polymer onto the antigen surface, forming a stable protective layer [21]. Studies involving positively charged polymers, such as chitosan or polyethyleneimine, have shown that these polymers enhance antigen stability under physiological conditions. Additionally, they improve the efficiency of antigen uptake by dendritic cells [22]. The polymer molecular weight and chain architecture also play a crucial role in influencing the steric hindrance effect, which further impacts antigen binding and protection. Increasing PEG molecular weight or employing branched PEG structures enhances the steric hindrance effect of the polymer shell, effectively shielding antigen enzymatic cleavage sites and prolonging drug half-life [20,23]. Responsive polymers have also been explored to improve antigen protection under variable environmental conditions, such as acidic or enzyme-rich microenvironments [14]. Furthermore, polymeric nanospheres, with varying polymer types, copolymer compositions, microsphere sizes, and morphologies, can physically shield the encapsulated components from external environmental interference while enabling controlled release, and have already been applied in drug delivery systems [24,25,26]. Although these studies demonstrate progress in polymer-based antigen capture and enzymatic protection, most efforts remain focused on novel material development and design, with limited systematic investigation into the molecular mechanisms governing polymer–antigen interactions and scarce quantitative comparison of polymer performance. In contrast, the present study combines computational simulations and experimental validation to elucidate these mechanisms and quantitatively evaluate the binding efficiency of different polymers, providing guidance for the rational design of polymers for in situ cancer vaccines.

This study aims to address this gap by investigating how polymer composition and degree of polymerization (DP) govern antigen capture, stabilization, and resistance to enzymatic degradation. The binding process between polymers and the tumor-specific antigen MAGE-A3 was investigated using molecular dynamics simulations and experimental methods. This study elucidates the interaction mechanisms between polymers, antigens, and hydrolases, clarifying the contributions of steric hindrance and electrostatic interactions to antigen enrichment and stability. Using BCA protein quantification assays and atomic force microscopy (AFM) measurements, we quantitatively investigate and evaluate the antigen capture efficiency of polymers, as well as the interactions between MAGE-A3 antigen and polymers of varying types and DPs. This approach reveals how polymer composition and DP influence antigen-binding affinity, capture efficiency, and resistance to enzymatic degradation.

## 2. Materials and Methods

**Figure 1:**
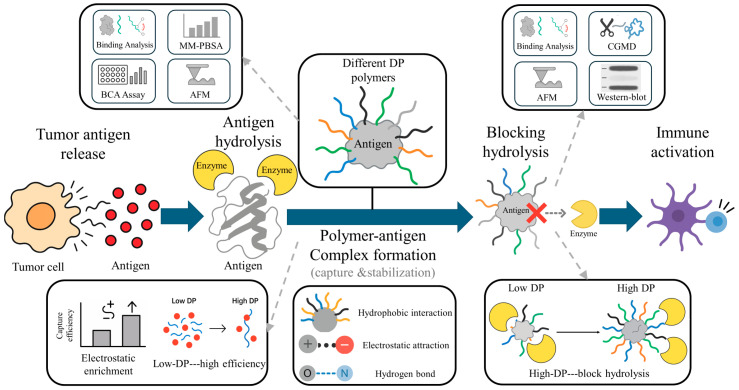
Workflow illustrating the biological process and analytical strategy of this study. Tumor antigens released from tumor cells interact with polymers of different DPs to form stabilized complexes. The upper panels summarize the simulation and experimental approaches, while the lower panels outline the revealed mechanisms, showing that polymer–antigen binding involves hydrogen bond, hydrophobic and electrostatic interactions, and that both polymer structure and DP collectively determine antigen capture efficiency and hydrolysis blocking capacity. In the “Blocking hydrolysis” panel, the red cross indicates the prevention of direct contact between the antigen and the enzyme. The gray dashed arrows indicate that the illustrated mechanisms or experimental methods originate from specific steps of the study workflow. This workflow illustration was manually created using Microsoft PowerPoint.

To systematically elucidate the mechanism by which polymers capture antigens after tumor antigen release and stabilize them, the research workflow is illustrated in Figure 1:

### 2.1. Materials

Considering that the structural composition of different polymers influences their mechanisms of action, this study selected five representative natural and synthetic polymers, including polysaccharides (chitosan, sodium alginate), polyesters (PCL, PLGA), and polyamines (PEI). These materials represent distinct structural types and physicochemical properties: polysaccharides are frequently used for antigen binding and delivery due to their excellent biocompatibility and charge characteristics; polyesters are widely applied in drug sustained release owing to their degradability and hydrophobicity; while polyamines enhance electrostatic interactions with antigens through their high-density positive charge. By systematically comparing the interactions of these polymers with antigens, we elucidate the differential patterns governing their antigen capture efficiency and stability regulation.

The polymers used in this study include: Chitosan (9012-76-4, MW = 100,000; Mw: 800–1000), Polycaprolactone (PCL, 24980-41-4, average Mn 80,000; average Mn 2000), polyethyleneimine (PEI, CAS No. 9002-98-6, M.W. 100,000, 35% aqueous solution; M.W. 3000, 25% aqueous solution), sodium alginate (ALGIN, CAS No. 9005-38-3, M/G = 1:2, molecular weight: 100 K), all purchased from McLean Reagents Co., Ltd. (Shanghai, China). Poly (lactic-co-glycolic acid) (PLGA, 34346-01-5, 50/50, Mw = 100,000; Mw = 5000) was purchased from Guangzhou Weihua Biotechnology Co., Ltd., Guangzhou, China. The BCA protein quantification kit was purchased from Shanghai Paisenuo Biotechnology Co., Ltd., Shanghai, China.

### 2.2. Molecular Dynamic Simulations

To investigate polymer–antigen binding and polymer-mediated inhibition of antigen–enzyme interactions, both all-atom (AA) and coarse-grained (CG) molecular dynamics models were constructed. The AA simulations were designed to examine how the MAGE-A3 antigen interacts with different polymers, whereas the CG simulations were used to determine how polymers impede antigen–enzyme association and protect antigens from hydrolysis. The MAGE-A3 antigen structure was obtained from the Protein Data Bank (PDB ID: 4V0P) [27,28], and the system was constructed and simulated on the GROMACS platform [29] with the AMBER99SB force field [30]. Representative polymer models with different DPs (40, 200, 1000) were generated using molecular modeling tools [31,32,33,34]. These DP values were selected to represent low-, intermediate-, and high-chain-length regimes across different polymer classes, thereby enabling a normalized mechanistic comparison with the experimentally used polymers, which span a broad molecular-weight range. The MAGE-A3 antigen and polymers were placed in equal masses within a simulated environment that contained SPC/E water molecules [35], and the system subsequently underwent 50 ns of MD simulations. A convergence analysis was performed for the last 20 ns of each simulation by calculating the binding free energy (${\Delta G}_{\mathrm{bind}}$) at 5 ns intervals, as shown in Figure S1, confirming that the polymer–protein interactions reached equilibrium within the simulated timeframe. Regulation of antigen–enzyme interactions by polymers was evaluated by constructing a coarse-grained system that comprised MAGE-A3 and Carboxypeptidase A1 (PDB ID: 2V77) [36], generated using the Martinize2 [37] and Martini3 force fields [38], while polymer parameters were optimized with AutoFF [39]. The system was simulated under standard periodic boundary conditions and long-range electrostatic interactions were handled through the PME method [40], whereas all remaining simulation parameters followed established conventions [41,42,43]. All simulations were conducted in triplicate to ensure statistical reliability.

Visualization of simulation results [44,45,46] and conformational analysis were carried out to evaluate the structure of the polymer–antigen systems. Protein structural stability was assessed through the root-mean-square deviation (RMSD) and the radius of gyration (Rg), whereas binding compactness was examined through the mindist algorithm that determines the number of contacting atoms when the interatomic distance is less than 0.6 nm. The buried surface area at the antigen–polymer interface (${BSA}_{\mathrm{total}}$) was obtained indirectly from the solvent-accessible surface area (*SASA*) as defined in Equation (1):

(1) $${BSA}_{\mathrm{total}}\;=\;\left({SASA}_{\mathrm{antigen}}\;+\;{SASA}_{\mathrm{polymer}}\right)\;-\;{SASA}_{\mathrm{complex}}$$

The solvent-accessible surface areas ${SASA}_{\mathrm{antigen}}$ and ${SASA}_{\mathrm{polymer}}$ represent those of the individual antigen and polymer, respectively, while the solvent-accessible surface area of the complex is denoted as ${SASA}_{\mathrm{complex}}$.

### 2.3. Binding Free Energy Calculation

The binding free energy between the MAGE-A3 antigen and polymers reflects the intrinsic strength of their interactions and the stability of the resulting complexes. Quantitative characterization of the binding capacity of different polymers in antigen capture was achieved through binding free energy calculations that were performed with the gmx_mmpbsa tool [47] on equilibrated molecular dynamics trajectories. The binding free energy (${\Delta G}_{\mathrm{bind}}$) is defined in Equations (2)–(4), where the total value is expressed as the combined contribution of Coulombic interactions, the Lennard–Jones potential in vacuum (${\Delta G}_{\mathrm{gas}}$), and the solvation energy (${\Delta G}_{\mathrm{sol}}$).

(2) $${\Delta G}_{\mathrm{bind}}={\Delta G}_{\mathrm{gas}}+{\;\Delta G}_{\mathrm{sol}}-T\Delta S$$

This comprises electrostatic interactions (${\Delta G}_{\mathrm{elec}}$) and van der Waals forces (${\Delta G}_{\mathrm{vdw}}$); ${\Delta G}_{\mathrm{sol}}$ comprises the polar electrostatic solvation free energy (${\Delta G}_{\mathrm{PB}}$) and the nonpolar solvation energy (${\Delta G}_{\mathrm{np}}$):

(3) $${\Delta G}_{\mathrm{gas}}\;=\;{\Delta G}_{\mathrm{elec}}\;+{\;\Delta G}_{\mathrm{vdw}}$$

(4) $${\Delta G}_{\mathrm{sol}}={\;\Delta G}_{\mathrm{PB}}+{\Delta G}_{\mathrm{np}}$$

Since the entropy term (TΔS) contributes only marginally in most protein–polymer systems, its effect is omitted in the present analysis [48]. Lower binding free energy values indicate greater stability of the antigen–polymer complex. In the calculations, snapshots were extracted every 100 ps from equilibrated MD trajectories so that energy decomposition and statistical averaging could be carried out with adequate numerical reliability. The resulting values were then correlated with conformational parameters through subsequent analysis that clarified how different interaction energies contribute to polymer–antigen capture.

### 2.4. Characterization of Antigen Capture In Vitro

The ability of polymers to capture tumor antigens in vitro reflects their suitability for application in situ cancer vaccines and provides experimental evidence that supports the binding energies and interfacial contact levels obtained from molecular dynamics simulations. A systematic evaluation of the antigen-binding performance of different polymers was carried out through an antigen-capture system that was constructed on the basis of tumor cell lysates. Esophageal squamous cell carcinoma (ESCC) cells (KYSE-150; Wuhan Sunncell Biotechnology Co., Ltd., Wuhan, China) that express high levels of the tumor-specific antigen MAGE-A3 were suspended in cold deionized water and subjected to five cycles of liquid-nitrogen freeze–thaw treatment [17] so that mechanical and osmotic damage would induce complete cell lysis and release intracellular tumor-associated antigens. The resulting lysate was centrifuged at 5000 rpm for 30 min through a 50 kDa ultrafiltration membrane so that cellular debris and macromolecular impurities were removed and a soluble antigen source was obtained. Total protein concentration was then measured with a BCA protein quantification kit and adjusted to a uniform initial level so that different experimental groups could remain comparable. Polymer solutions were prepared at 100 μg/mL for nine systems that included chitosan, PCL, PEI, and PLGA at both low and high DPs together with sodium alginate at a high DP. The concentration of 100 μg/mL was selected as a unified condition for comparative evaluation and was chosen with reference to previous in vitro antigen-capture studies, including PEI-based antigen-capture systems. Each polymer solution was subsequently mixed with the MAGE-A3 antigen solution (6 mg/mL) and incubated at 37 °C for 4 h so that antigen capture under physiological conditions could be simulated. Following incubation, the mixtures were reprocessed through a 50 kDa ultrafiltration membrane (5000 rpm, 30 min). After incubation, the mixtures were processed again through a 50 kDa ultrafiltration membrane (5000 rpm, 30 min), and the filtrate was collected for absorbance measurements at 343 nm. Residual antigen concentrations were obtained from a BCA calibration curve, and quantitative capture efficiencies were derived by comparison with an untreated control. Each polymer system was evaluated through three independent replicates, and the resulting data were analyzed through one-way analysis of variance so that significant differences across polymer categories and DPs could be identified. Because the lysate-based BCA assay reflects overall protein/antigen retention rather than strict MAGE-A3-specific quantification, the present experimental results should be interpreted as trend-level support for the mechanistic findings obtained from the single-antigen simulation model.

### 2.5. Interaction Force Measurements

AFM single-molecule force spectroscopy enables quantitative measurement of antigen–polymer binding forces at the molecular scale and directly reveals the strengths of these interactions. The binding characteristics of different polymers toward the antigen were examined through separately prepared and functionalized antigen probes and polymer substrates. The AFM cantilever (spring constant 80–100 pN/nm, frequency 12–24 kHz) was first treated with a plasma cleaner for 10 min so that surface contaminants could be removed [49]. The treated cantilever was then incubated for 1 h in 50 μL of MAGE-A3 solution (10 μg/mL) so that the antigen protein could adsorb onto the tip surface. After air-drying, the functionalized probes were stored at 5 °C until use to maintain their stability. For preparation of polymer substrates, nine polymers were dissolved to 1 mg/mL, with chitosan, PEI, and sodium alginate dissolved in deionized water and PCL and PLGA dissolved in dichloromethane. A volume of 200 μL of each polymer solution was dispensed onto a clean Petri dish and gently swirled so that uniform spreading could be achieved, after which the dish was left to air-dry and the resulting solid coating was stored at 5 °C for later use.

AFM measurements were carried out on an MFP-3D-BIO instrument under controlled and standardized operating conditions. The experimental parameters were set with an indentation and retraction distance of 0.5 μm, a scanning rate of 0.5 Hz, a dwell time of 1 s, and a trigger force of 1 nN. Three independent samples were prepared for each polymer, and measurements were obtained with three separately functionalized probes so that variations arising from probe fabrication could be minimized. Each sample was examined at nine randomly selected positions, and nine force curves were recorded at each position, resulting in a total of 729 curves that provided adequate statistical robustness. Probe-modification stability was verified through reference measurements on a glass substrate before and after each experiment so that consistency in antigen functionalization could be confirmed. The resulting force-curve distributions were analyzed through Gaussian fitting. To distinguish non-specific adhesion from specific antigen–polymer interactions, blank AFM measurements were performed in the absence of polymer or antigen. A representative force–distance curve and the distribution of minimum forces obtained from these controls are presented in Figure S4.

Additionally, the inhibitory effect of polymers on antigen–enzyme binding was evaluated through experiments that employed the enzyme as a substrate. A carboxypeptidase solution was spread uniformly onto a clean Petri dish and left to air-dry so that an enzyme-modified substrate could be formed. Force measurements were carried out with untreated antigen probes and polymer-modified antigen probes on this substrate under identical operating procedures and parameter settings. Force–distance curves obtained from these measurements were analyzed to determine the extent to which polymer coating weakened antigen–enzyme interactions.

### 2.6. Validation of Antigen Protection In Vitro

Western blot (WB) analysis was used to validate the protective role of polymers by assessing antigen stability during enzymatic digestion. Two experimental groups were prepared: a control group without polymer treatment and an experimental group in which the antigen was pre-coated with high-DP chitosan. A MAGE-A3 protein solution (10 μg/mL) was incubated with a carboxypeptidase solution at 37 °C for 2 h so that enzymatic digestion could proceed under controlled conditions. For the experimental group, the antigen was mixed with a 100 μg/mL high-DP chitosan solution for 1 h before incubation so that polymer coating could be achieved. Immediately after incubation, SDS-PAGE loading buffer was added so that the reaction could be terminated.

After separation by 10% SDS-PAGE, the sample was transferred to a PVDF membrane (Millipore) and blocked in 5% nonfat dry milk TBST solution for 1 h. Primary antibody (anti-MAGE-A3 polyclonal antibody, diluted 1:1000) was added and incubated overnight at 4 °C. After three washes with TBST, HRP-labeled secondary antibody (diluted 1:5000) was added and incubated at room temperature for 1 h. The membrane was developed using ECL chemiluminescent reagent, and bands were recorded with an imaging system. The protective effect of high-polymerization chitosan against antigen hydrolysis was evaluated by comparing the intensity changes in intact MAGE-A3 protein bands between the control and experimental groups.

## 3. Results and Discussion

### 3.1. Effectiveness of Antigen Capture by Polymers

**Figure 2:**
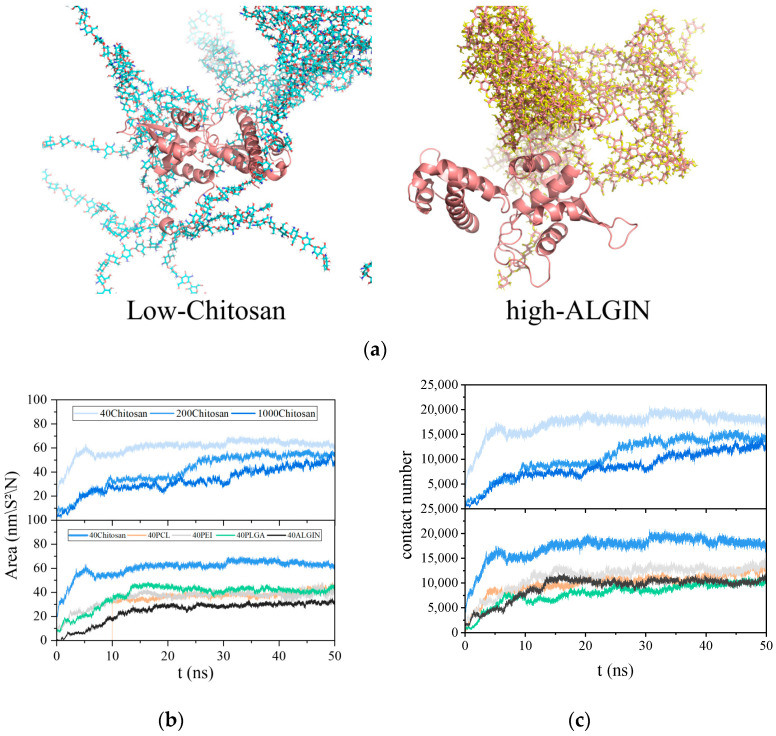
Representative conformational and interfacial analyses of MAGE-A3–polymer complexes. (**a**) Representative snapshots of antigen–polymer binding configurations. The antigen is shown as a salmon-colored cartoon, while the polymers are shown in stick representation; (**b**) Time-dependent changes in buried surface area (BSA); (**c**) Number of contact atoms between antigen and polymer over simulation time. Darker colors indicate higher degrees of polymerization. Blue, orange, gray, green, and black represent chitosan, PCL, PEI, PLGA, and alginate, respectively.

**Figure 3:**
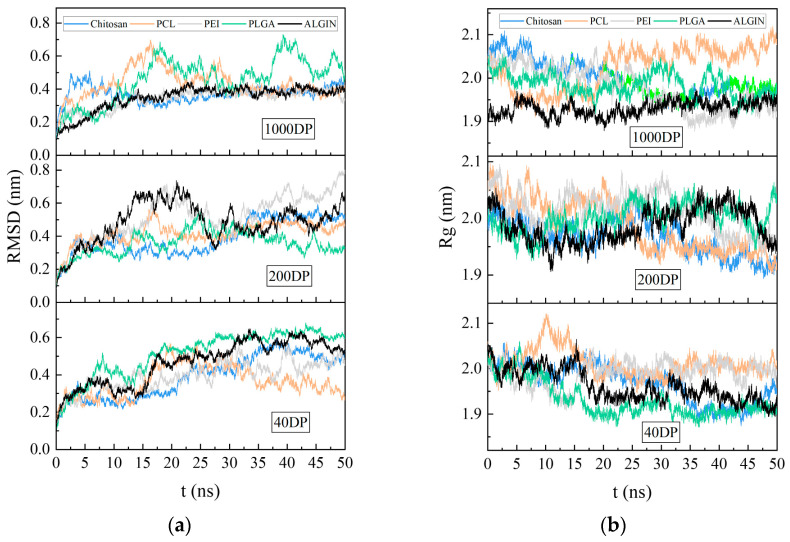
Stability analyses of MAGE-A3–polymer complexes. (**a**) RMSD of the antigen; (**b**) Rg of the antigen. Blue, orange, gray, green, and black represent chitosan, PCL, PEI, PLGA, and alginate, respectively.

The number of binding atoms and the buried surface area (BSA) between antigens and polymers serve as key quantitative indicators that reflect the capacity of polymers to capture antigens. Figure 2a illustrates that different polymers exhibited distinct binding efficiencies toward the MAGE-A3 antigen. Chitosan formed a dense coating on the antigen surface through molecular segments that adhered tightly to extensive structural regions and maintained stable interlocking. In contrast, sodium alginate produced more dispersed contacts with smaller interacting regions on the antigen surface. The chitosan system sustained the highest BSA value throughout the simulation, as shown in Figure 2b, and the value eventually reached 60.324 nm^2^. PEI, PCL, and PLGA followed in decreasing order, whereas sodium alginate displayed slightly lower values. The variation curves of contacting atoms, illustrated in Figure 2c, showed that chitosan also maintained the highest number of contacting atoms at approximately 15,000, whereas PLGA and sodium alginate exhibited the lowest levels. These observations indicate that chitosan reduces the exposed surface area of the antigen effectively through stable surface binding. As shown in Figure 3a,b, the RMSD of the antigen in all polymer systems remained within the range of 0.2 to 0.8 nm without sustained increases over the course of the simulation, which suggests that polymer binding did not induce protein unfolding. The Rg fluctuated within a narrow range around its equilibrium value and showed no indication of significant contraction or expansion.

The capture efficiency of polymeric antigens shows a clear negative correlation with the polymerization degree, as presented in Figure 2b,c. When the degree of polymerization increased to 1000, the BSA of the chitosan system decreased from 60.324 nm^2^ to 47.874 nm^2,^ and the number of atoms in contact with the antigen dropped from 16,832 to 12,660. This decrease may arise from the tendency of highly polymerized chains to undergo self-aggregation driven by enhanced self-wrapping and polymer–polymer interactions rather than spreading across the antigen surface. RMSD and Rg values in Figure 3a,b fluctuated within narrow ranges across the three polymerization levels and indicated that polymerization degree did not cause significant changes in antigen conformational stability. At lower polymerization levels, more complete interfacial coverage together with a more uniform distribution of contact sites produced smaller transient fluctuations in RMSD and Rg. These observations demonstrate the superior antigen-capturing performance of low-DP polymers.

**Figure 4:**
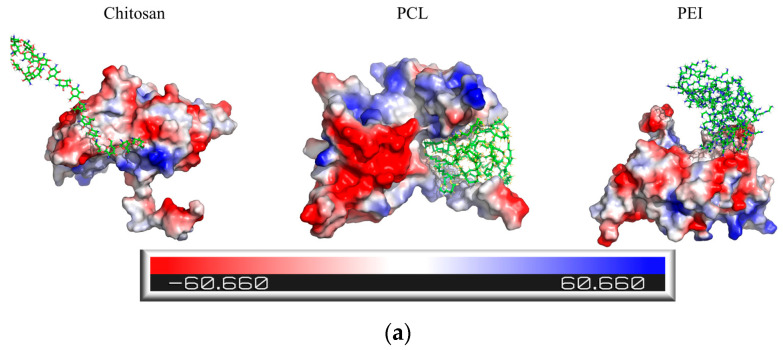

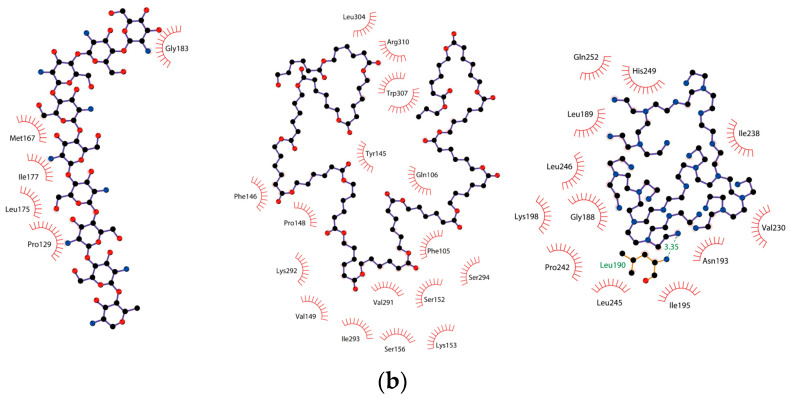
Binding modes and energy decomposition analyses of polymer–antigen complexes. (**a**) Electrostatic potential surface of MAGE-A3 antigen and polymer-binding regions. The molecular surface is colored according to electrostatic potential, ranging from −60.66 to 60.66 kT/e, with red indicating negative potential and blue indicating positive potential; (**b**) Two-dimensional LigPlot diagrams of polymer–antigen interactions. Black, red, and blue circles represent carbon, oxygen, and nitrogen atoms, respectively; green dashed lines indicate hydrogen bonds, and red spoked arcs indicate hydrophobic interactions.

To further investigate the binding mechanism between the antigen and the polymers, the interfacial binding sites were visualized and analyzed, as shown in Figure S2. Electrostatic potential surface mapping in Figure 4a revealed extensive negatively charged regions (red) on the MAGE-A3 antigen surface, and these regions facilitated the selective adsorption of positively charged polymers. Chitosan and PEI formed stable electrostatic interactions with these negatively charged areas and displayed clear electrostatically driven binding behavior. In contrast, PCL interacted mainly with antigen regions that contained both positive and negative charges. LigPlot diagrams in Figure 4b further illustrated the structural characteristics of the binding interface. Hydrophobic interactions were detected between all polymers and the antigen, and these observations suggest that hydrophobic forces act as the primary driving mechanism for polymer–antigen association. Among the examined systems, PEI additionally formed hydrogen bonds with multiple antigen residues and strengthened interfacial stability beyond the contribution from hydrophobic interactions.

**Figure 5:**
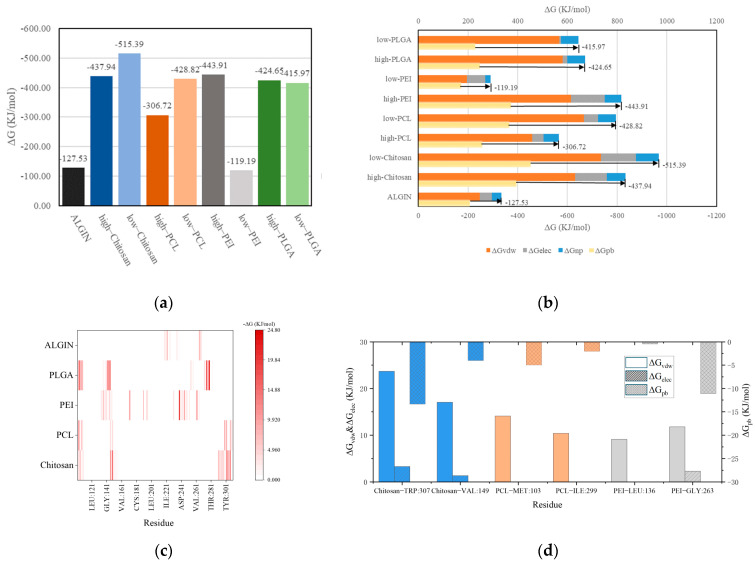
Binding modes and energy decomposition analyses of polymer–antigen complexes. (**a**) Binding free energies (${\Delta G}_{\mathrm{bind}}$) of different polymer systems; (**b**) Energy component decomposition including van der Waals, electrostatic, polar solvation, and non-polar solvation terms. The arrow indicates the total ${\Delta G}_{\mathrm{bind}}$ obtained from the sum of all plotted energy components, which is consistent with the ${\Delta G}_{\mathrm{bind}}$ value shown in Figure 5a; (**c**) Residue-based energy contribution maps for representative polymer systems; (**d**) Residue-based energy decomposition of representative polymer–antigen systems.

The binding free energy calculations in Figure 5a revealed marked differences in affinity among the polymers. Low-DP chitosan exhibited the lowest binding free energy (−515.39 kJ/mol) and therefore showed the strongest binding affinity, which is consistent with its high BSA and large number of contacting atoms. High-DP PEI and chitosan also displayed relatively low binding free energies (−443.91 and −437.94 kJ/mol, respectively) and demonstrated the distinct advantage of cationic polymers in antigen capture. In contrast, the binding free energies of sodium alginate and low-DP PEI were approximately −120 kJ/mol and indicated low binding stability. Energy-decomposition results in Figure 5b revealed the contribution profiles of the different interaction types. Van der Waals interactions (${\Delta G}_{\mathrm{vdw}}$) acted as the universal driving force in all systems and were particularly dominant in PCL and PLGA, indicating that hydrophobic polymers depend on nonpolar interactions to maintain binding. Electrostatic interactions (${\Delta G}_{\mathrm{elec}}$) contributed primarily in the PEI and chitosan systems and correlated strongly with their high charge densities. Polar solvation energy (${\Delta G}_{\mathrm{PB}}$) weakened polymer–antigen interactions, whereas nonpolar solvation energy (${\Delta G}_{\mathrm{np}}$) showed smaller absolute values across all polymers but still played a supplementary role in hydrophobic systems.

Residue-level energy decomposition further revealed the interaction profiles of the key amino acid residues located at the antigen interface. Figure 5c presents a heatmap of residue energy contributions across the different polymer systems. The primary interacting residues in the PCL and chitosan systems were distributed in relatively similar regions, whereas PEI and alginate exhibited almost completely distinct residue-interaction patterns and reflected substantial differences in binding modes. Figure 5d identifies the major contributing residues involved in binding within the chitosan, PCL, and PEI systems. Hydrophobic interactions dominated by ${\Delta G}_{\mathrm{vdw}}$ served as the primary driving force for binding, and PCL showed negligible contributions from electrostatic interactions. In contrast, ${\Delta G}_{\mathrm{elec}}$ contributed significantly among the active residues in the chitosan and PEI systems. Notably, ${\Delta G}_{\mathrm{elec}}$ and the sterically hindered ${\Delta G}_{\mathrm{PB}}$ exhibited a positive correlation and suggested that although electrostatic interactions were strong, they remained restricted by hydration effects.

**Figure 6:**
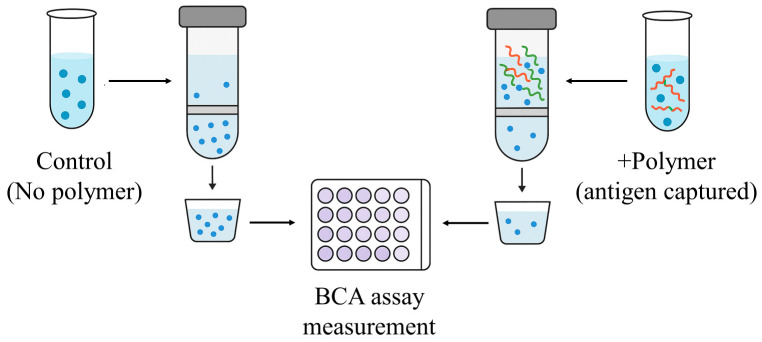
Schematic diagram of the BCA assay workflow. The curved lines denote the polymer chains, while the blue dots denote the antigen proteins.

**Figure 7:**
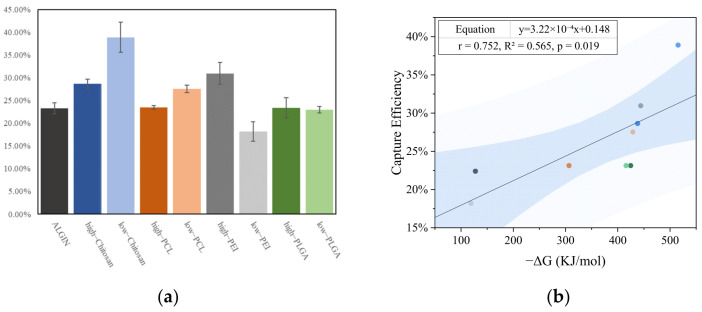
In vitro antigen capture assay and correlation with simulation results. (**a**) Antigen capture efficiencies of different polymer systems; (**b**) Correlation between binding free energies (${\Delta G}_{\mathrm{bind}}$) obtained from simulations and antigen capture efficiencies. The dot colors follow the same polymer-type assignments as in Figure 7a. The two blue bands denote the 95% confidence band and the prediction band, respectively.

The antigen-capturing abilities of different polymers were quantified through BCA protein assays so that the accuracy of the preceding simulation analyses could be validated. The experiment was performed on the basis of ultrafiltration separation and colorimetric quantification, as illustrated in Figure 6. The results revealed substantial differences in antigen-capture performance among the polymers, as shown in Figure 7a. Low-DP chitosan exhibited the highest capture efficiency with a value approaching 40%. High-DP PEI also achieved a capture efficiency exceeding 30%, whereas low-DP PEI and sodium alginate showed markedly lower values. Other polymers, including PCL and PLGA, exhibited capture efficiencies distributed within the range of 20–25%. These measurements demonstrated a trend consistent with the simulation data. Furthermore, Figure 7b shows a strong linear correlation between the binding free energies obtained from simulation and the capture efficiencies measured through BCA assays and confirms the reliability of the computational predictions.

**Figure 8:**
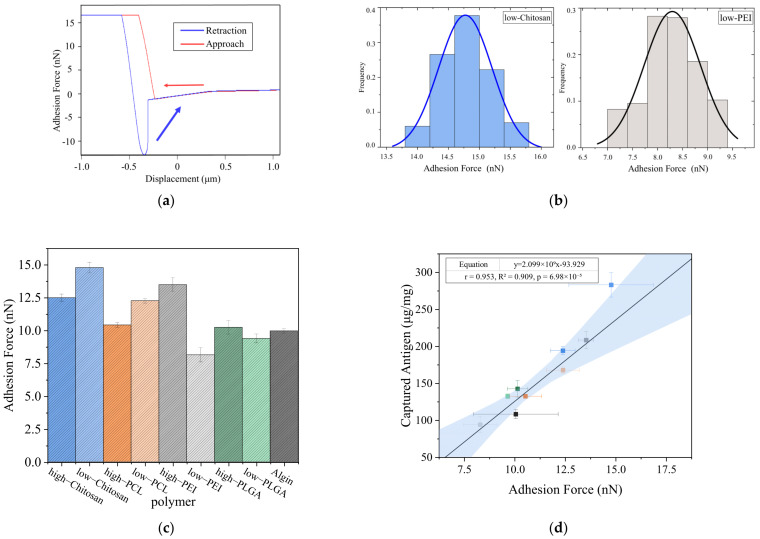
Single-molecule force spectroscopy analysis of polymer–antigen interactions. (**a**) Representative AFM force–distance curve. The arrows indicate the directions of the AFM tip during approach to and retraction from the sample; (**b**) Adhesion force distributions of low-degree chitosan and PEI systems; (**c**) Average adhesion forces of different polymer systems; (**d**) Correlation between adhesion forces and antigen capture efficiencies. The dot colors correspond to the same polymer types as those in Figure 8c. The two blue bands represent the 95% confidence band and the prediction band, respectively.

Additionally, AFM measurements revealed characteristic desorption peaks that appeared clearly in individual force–distance curves, as shown in Figure 8a, and reflected the strengths of the molecular interactions. Statistical evaluation of numerous single-molecule force curves in Figure S3 showed that the adhesion-force distribution between low-DP chitosan and the antigen ranged from 14 to 15.5 nN, whereas the distribution for low-DP PEI remained significantly lower within 7 to 9.5 nN, as presented in Figure 8b. This discrepancy indicated that chitosan formed stronger interactions with the antigen, whereas low-DP PEI exhibited insufficient binding due to its extremely low molecular weight. When the average adhesion forces were compared across the polymer systems in Figure 8c, low-DP chitosan reached approximately 15 nN and showed the highest value among all systems. High-DP PEI achieved an average adhesion force of about 12.5 nN and displayed strong adsorption, whereas low-DP PEI exhibited the weakest binding strength with average values below 8.5 nN. PCL, PLGA, and sodium alginate demonstrated intermediate adhesion strengths of around 10 nN. Furthermore, the AFM adhesion-force measurements showed a strong linear correlation with the capture efficiencies obtained from the BCA assays in Figure 8d and confirmed that the AFM results closely matched the simulated binding trends.

### 3.2. Inhibitory Effect of Polymers on Antigen-Enzyme Interactions

Whether polymers can effectively prevent the interaction between antigens and hydrolases is a critical factor determining their protective function in in situ antigen-capture vaccines. To investigate this mechanism, a simulation system containing the MAGE-A3 antigen and carboxypeptidase was established, into which different polymers were introduced for coarse-grained molecular dynamics simulations to elucidate how steric hindrance impedes antigen–enzyme binding. In parallel, atomic force microscopy (AFM) and Western blot experiments were performed to validate the polymers’ efficacy in blocking antigen–enzyme interactions.

**Figure 9:**
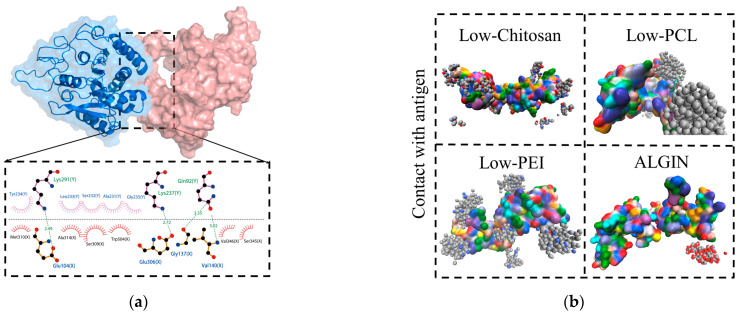

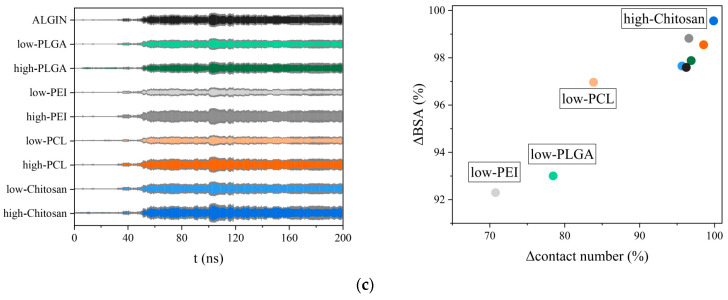
Coarse-grained molecular dynamics simulations of polymer-mediated inhibition of antigen–enzyme interactions. (**a**) Representative snapshots from all-atom MD simulations of MAGE-A3 antigen binding with carboxypeptidase. The salmon-colored structure represents the antigen, and the blue structure represents the enzyme carboxypeptidase. Black, red, and blue circles represent carbon, oxygen, and nitrogen atoms, respectively; green dashed lines indicate hydrogen bonds, and red spoked arcs indicate hydrophobic interactions; (**b**) Coarse-grained simulation snapshots of different polymer systems illustrating encapsulation and shielding effects. The antigen protein is shown in surface representation, while the polymers are shown in sphere representation; (**c**) Kite and scatter plots showing polymer-mediated inhibition of antigen–enzyme binding.

Figure 9a shows that the antigen and the enzyme remained in close contact in the control system and that the antigen was highly exposed to the enzymatic cleavage sites, which led to degradation. LigPlot analysis supported this observation and revealed hydrogen bonds formed between Lys and Glu residues in both groups, together with hydrophobic interactions involving Leu, Ser, Ala, and Gly residues that indicated stable antigen–enzyme interactions. After polymer introduction, direct antigen–enzyme binding was markedly weakened, as shown in Figure 9b, and the complete set of results is provided in Figure S5. Charged polymers such as chitosan and PEI adhered tightly to the antigen surface through electrostatic interactions and thereby reduced enzyme–antigen contacts. Chitosan exhibited the strongest inhibitory effect, whereas PEI also formed a stable electrostatic barrier at the interface. The hydrophobic polymer PCL adhered to the antigen primarily through nonpolar interactions and provided effective surface coverage. In contrast, sodium alginate showed the weakest shielding effect because of its dispersed binding pattern. Quantitative analyses in Figure 9c corroborated these observations and showed that the chitosan system produced the widest range in the kite plot of surface-obstructed contact area and occupied the upper-right quadrant in the scatter plot constructed from BSA and contact-atom counts, which demonstrated its superior ability to reduce both contact atoms and BSA.

The polymerization degree and molecular weight exerted a critical influence on the inhibitory effect against antigen–enzyme binding. High-polymerization polymers generated substantial steric hindrance because of their large molecular volume and prevented direct antigen–enzyme contact effectively. Although low-polymerization polymers adhered well to the antigen surface in the simulations and partially encapsulated both the antigen and the enzyme, their shorter chain lengths produced thinner coating layers. As a result, the enzyme could still penetrate the interfacial gaps and access the antigen surface. This limitation was particularly evident for low-DP PEI, whose small molecular weight produced discontinuous surface coverage and hindered the formation of a complete spatial barrier. Quantitative analyses in Figure 9c corroborated these observations and showed that high-DP chitosan blocked 164 antigen–enzyme contact atoms, whereas low-DP chitosan blocked 130 contact atoms. In the scatter plots, the high-polymerization systems occupied the upper-right region and indicated the strongest blocking capacity, whereas the low-polymerization systems clustered in the region associated with weaker inhibitory performance.

**Figure 10:**
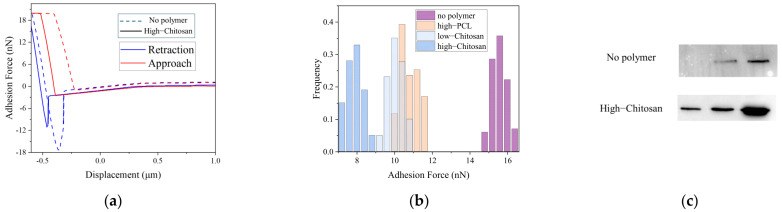
Experimental validation of polymer-mediated blocking of antigen–enzyme interactions. (**a**) Representative force–distance curves of control and high-degree chitosan groups. The dashed and solid lines denote the experiments without polymer and with high-DP chitosan, respectively; (**b**) Adhesion force distributions of different polymer systems; (**c**) Western blot analysis of antigen integrity under enzymatic hydrolysis.

The preceding simulation results were further validated experimentally. AFM measurements obtained with polymer-loaded antigen probes revealed force–distance curves that showed reduced binding forces in the presence of polymers, as presented in Figure 10a. In the polymer-free control group, the peak adhesion force between the antigen and the enzyme reached approximately 15 nN. After high-DP chitosan was loaded onto the antigen, the adhesion force decreased to about 8 nN, demonstrating that polymer coating markedly weakened antigen–enzyme interactions. The distribution of adhesion forces further revealed differences among the polymers in Figure 10b. Charged polymers produced stronger blocking effects than hydrophobic polymers, and high-DP chitosan exhibited the most pronounced inhibition. Low-DP chitosan and PCL also reduced antigen–enzyme binding forces. Overall, the AFM measurements showed trends consistent with the simulation results.

Western blot (WB) experiments yielded results consistent with the AFM measurements, as shown in Figure 10c. In the polymer-free control group, the MAGE-A3 antigen exhibited clear degradation bands after incubation with carboxypeptidase, and the intensity of the intact antigen band decreased markedly, which indicated high susceptibility to enzymatic hydrolysis. In contrast, the intact antigen band remained clearly visible in the group treated with high-molecular-weight chitosan, and the extent of degradation was substantially reduced. These results confirmed that the polymer prevented enzyme–antigen contact through steric hindrance and significantly delayed the hydrolysis process.

These results suggest that practical in situ vaccine design may require balancing antigen-capture efficiency and hydrolysis protection, rather than maximizing either factor alone. In particular, they reveal that low-DP and high-DP polymers play distinct but complementary roles in antigen retention and stabilization, providing mechanistic guidance for the design of capture-protection integrated systems.

## 4. Conclusions

The regulatory effects of polymer type and DP on the antigen-capturing and anti-hydrolytic performance of the tumor-specific antigen MAGE-A3 were elucidated through molecular dynamics simulations. The charge density and molecular structure of a polymer modulate its electrostatic and hydrophobic interactions with the antigen and thereby alter its binding force toward the antigen. Polymers bind to antigens primarily through van der Waals interactions, and cationic polymers further enhance this binding through electrostatic adsorption. Compared with their high-DP counterparts, low-DP polymers increased the average number of antigen–polymer contact atoms by 32.9% and the average buried surface area (BSA) by 26.0%, indicating stronger surface spreading and interfacial coverage. However, high-DP polymers have large molecular dimensions that produce strong steric hindrance, and this steric hindrance increases their resistance to enzymatic hydrolysis. Compared with low-DP systems, high-DP polymer systems reduced the average number of antigen–enzyme contact atoms by 26.2%, reflecting their stronger steric shielding effect. Chitosan with a polymerization degree of 200 showed effective performance in both antigen capture and resistance to hydrolysis. Experimental observations led to conclusions that were consistent with the simulation results. Compared with the neutral polymers PCL and PLGA and the anionic polymer alginate, the cationic polymers PEI and chitosan showed stronger binding to the antigen. Among these polymers, low-DP chitosan exhibited the highest antigen-capturing efficiency at 39.8%. This arises from the ability of its short-chain segments to interact with multiple sites on the antigen surface. In addition, high-DP chitosan can bind to the antigen and generate a strong steric hindrance that effectively blocks antigen–enzyme association. Compared with low-DP polymers, high-DP chitosan showed a 37.5% greater reduction in antigen–enzyme binding force, indicating superior blocking performance. These findings provide mechanistic guidance for optimizing polymer architecture and functional design, and they may facilitate future studies on integrating antigen capture, protection, and immune activation in in situ cancer vaccine systems.

## Data Availability

The raw data supporting the conclusions of this article will be made available by the authors on request.

## Supplementary Materials

The following supporting information can be downloaded at: https://www.mdpi.com/article/10.3390/polym18070781/s1, Figure S1: Convergence analysis of polymer–protein binding free energy (${\Delta G}_{\mathrm{bind}}$) for the last 20 ns of MD simulations; Figure S2: Snapshots, LigPlot diagrams, and electrostatic potential surface distributions showing the binding sites of five types of polymers with the antigen; Figure S3: AFM-measured adhesion force distribution curves for nine polymer systems of five types with different degrees of polymerization; Figure S4: AFM blank control for non-specific adhesion; Figure S5: representative simulation snapshots illustrating the inhibition of antigen–enzyme binding by the nine polymer systems.

## References

[1] Li W., Jing Z., Wang S., Li Q., Xing Y., Shi H., Li S., Hong Z. (2021). P22 virus-like particles as an effective antigen delivery nanoplatform for cancer immunotherapy. Biomaterials.

[2] Xue F., Liu J., Wu J., Li X., Zhu N., Tang S., Zhang M., Duan H., Wang R., Zhang J. (2025). Combination therapy with alisertib enhances the antitumor immunity induced by a liver cancer vaccine. iScience.

[3] Ding K., Mou P., Wang Z., Liu S., Liu J., Lu H., Yu G. (2023). The next bastion to be conquered in immunotherapy: Microsatellite stable colorectal cancer. Front. Immunol..

[4] Dariushnejad H., Ghorbanzadeh V., Akbari S., Hashemzadeh P. (2022). Design of a novel recombinant multi-epitope vaccine against triple-negative breast cancer. Iran. Biomed. J..

[5] Liu D., Che X., Wang X., Ma C., Wu G. (2023). Tumor vaccines: Unleashing the power of the immune system to fight cancer. Pharmaceuticals.

[6] Wang C., Xu L.G., Liang C., Xiang J., Peng R., Liu Z. (2014). Immunological responses triggered by photothermal therapy with carbon nanotubes in combination with anti-CTLA-4 therapy to inhibit cancer metastasis. Adv. Mater..

[7] Shen M., Du Y., Ye Y. (2022). Tumor-associated macrophages, dendritic cells, and neutrophils: Biological roles, crosstalk, and therapeutic relevance. Med. Rev..

[8] Kantoff P.W., Higano C.S., Shore N.D., Berger E.R., Small E.J., Penson D.F., Redfern C.H., Ferrari A.C., Dreicer R., Sims R.B. (2010). Sipuleucel-T immunotherapy for castration-resistant prostate cancer. N. Engl. J. Med..

[9] Chao C.J., Zhang E., Trinh D.N., Udofa E., Lin H., Silvers C., Huo J., He S., Zheng J., Cai X. (2025). Integrating antigen capturing nanoparticles and type 1 conventional dendritic cell therapy for in situ cancer immunization. Nat. Commun..

[10] Fobian S.F., Cheng Z., Ten Hagen T.L.M. (2021). Smart lipid-based nanosystems for therapeutic immune induction against cancers: Perspectives and outlooks. Pharmaceutics.

[11] Melief C.J., van Hall T., Arens R., Ossendorp F., van der Burg S.H. (2015). Therapeutic cancer vaccines. J. Clin. Investig..

[12] Danhier F., Ansorena E., Silva J.M., Coco R., Le Breton A., Préat V. (2012). PLGA-based nanoparticles: An overview of biomedical applications. J. Control. Release.

[13] Bo Y., Wang H. (2024). Biomaterial-based in situ cancer vaccines. Adv. Mater..

[14] Gong N., Alameh M.G., El-Mayta R., Xue L., Weissman D., Mitchell M.J. (2024). Enhancing in situ cancer vaccines using delivery technologies. Nat. Rev. Drug Discov..

[15] Liu X., Su Q., Song H., Shi X., Zhang Y., Zhang C., Huang P., Dong A., Kong D., Wang W. (2021). PolyTLR7/8a-conjugated, antigen-trapping gold nanorods elicit anticancer immunity against abscopal tumors by photothermal therapy-induced in situ vaccination. Biomaterials.

[16] Chen J., Su M., Xu C. (2024). Cationic lipid-polymer hybrid nanoparticle drives in situ generation and lymphatic navigation of tumor antigens to prime systemic antitumor immunity. Nano Today.

[17] Chen W., Zhang M., Wang C., Zhang Q. (2023). PEI-based nanoparticles for tumor immunotherapy via in situ antigen capture triggered by photothermal therapy. ACS Appl. Mater. Interfaces.

[18] Saleh M., El-Moghazy A., Elgohary A.H., Saber W.I.A., Helmy Y.A. (2025). Revolutionizing nanovaccines: A new era of immunization. Vaccines.

[19] Gutjahr A., Phelip C., Coolen A.L., Monge C., Boisgard A.S., Paul S., Verrier B. (2016). Biodegradable polymeric nanoparticles-based vaccine adjuvants for lymph node targeting. Vaccines.

[20] Shakibania S., Biggs M.J.P., Krukiewicz K. (2024). Adjusting cell-surface interactions through a covalent immobilization of biomolecules. Adv. Mater. Interfaces.

[21] Erol M., Du H., Sukhishvili S. (2006). Control of specific attachment of proteins by adsorption of polymer layers. Langmuir.

[22] Masimov R., Wasan E.K. (2024). Chitosan non-particulate vaccine delivery systems. J. Pharm. Pharm. Sci..

[23] Li C., Li T., Tian X., An W., Wang Z., Han B., Tao H., Wang J., Wang X. (2024). Research progress on the PEGylation of therapeutic proteins and peptides. Front. Pharmacol..

[24] Shen J., Burgess D.J. (2012). Accelerated in vitro release testing methods for extended-release parenteral dosage forms. J. Pharm. Pharmacol..

[25] Alavi M., Webster T.J. (2021). Recent progress and challenges for polymeric microsphere compared to nanosphere drug release systems: Is there a real difference?. Bioorg. Med. Chem..

[26] Jiang C., Kuang L., Merkel M.P., Yue F., Cano-Vega M.A., Narayanan N., Kuang S., Deng M. (2015). Biodegradable polymeric microsphere-based drug delivery for inductive browning of fat. Front. Endocrinol..

[27] Newman J.A., Cooper C.D., Roos A.K., Aitkenhead H., Oppermann U.C., Cho H.J., Osman R., Gileadi O. (2016). Structures of two melanoma-associated antigens suggest allosteric regulation of effector binding. PLoS ONE.

[28] Jurrus E., Engel D., Star K., Monson K., Brandi J., Felberg L.E., Brookes D.H., Wilson L., Chen J., Liles K. (2018). Improvements to the APBS biomolecular solvation software suite. Protein Sci..

[29] Abraham M.J., Murtola T., Schulz R., Páll S., Smith J.C., Hess B., Lindahl E. (2015). GROMACS: High performance molecular simulations through multi-level parallelism from laptops to supercomputers. SoftwareX.

[30] Lindorff-Larsen K., Piana S., Palmo K., Maragakis P., Klepeis J.L., Dror R.O., Shaw D.E. (2010). Improved side-chain torsion potentials for the Amber ff99SB protein force field. Proteins.

[31] Hanwell M.D., Curtis D.E., Lonie D.C., Vandermeersch T., Zurek E., Hutchison G.R. (2012). Avogadro: An advanced semantic chemical editor, visualization, and analysis platform. J. Cheminf..

[32] Wang J., Wolf R.M., Caldwell J.W., Kollman P.A., Case D.A. (2004). Development and testing of a general Amber force field. J. Comput. Chem..

[33] Lu T. (2025). Sobtop.

[34] Open Babel Development Team Open Babel. https://openbabel.github.io/index.html.

[35] Berendsen H.J.C., Grigera J.R., Straatsma T.P. (1987). The missing term in effective pair potentials. J. Phys. Chem..

[36] Pallarès I., Fernández D., Comellas-Bigler M., Fernández-Recio J., Ventura S., Avilés F.X., Bode W., Vendrell J. (2008). Direct interaction between a human digestive protease and the mucoadhesive poly(acrylic acid). Acta Crystallogr. D.

[37] Kroon P.C., Grunewald F., Barnoud J., van Tilburg M., Souza P.C.T., Wassenaar T.A., Marrink S.J. (2023). Martinize2 and Vermouth: Unified framework for topology generation. eLife.

[38] Souza P.C.T., Alessandri R., Barnoud J., Thallmair S., Faustino I., Grünewald F., Patmanidis I., Abdizadeh H., Bruininks B.M.H., Wassenaar T.A. (2021). Martini 3: A general-purpose force field for coarse-grained molecular dynamics. Nat. Methods.

[39] Wang C., Li W., Liao K., Wang Z., Wang Y., Gong K. (2023). AuToFF Program.

[40] Darden T., York D., Pedersen L. (1993). Particle mesh Ewald: An N·log(N) method for Ewald sums in large systems. J. Chem. Phys..

[41] Hirschfelder J.O., Curtiss C.F., Bird R.B. (1964). Molecular Theory of Gases and Liquids.

[42] Hess B., Bekker H., Berendsen H.J.C., Fraaije J.G.E.M. (1997). LINCS: A linear constraint solver for molecular simulations. J. Comput. Chem..

[43] Bussi G., Donadio D., Parrinello M. (2007). Canonical sampling through velocity rescaling. J. Chem. Phys..

[44] Humphrey W., Dalke A., Schulten K. (1996). VMD: Visual molecular dynamics. J. Mol. Graph..

[45] Laskowski R.A., Swindells M.B. (2011). LigPlot+: Multiple ligand–protein interaction diagrams for drug discovery. J. Chem. Inf. Model..

[46] Schrödinger, LLC (2020). PyMOL Molecular Graphics System.

[47] Valdés-Tresanco M.S., Valdés-Tresanco M.E., Valiente P.A., Moreno E. (2021). gmx_MMPBSA: A new tool to perform end-state free energy calculations with GROMACS. J. Chem. Theory Comput..

[48] Homeyer N., Gohlke H. (2012). Free energy calculations by the molecular mechanics Poisson–Boltzmann surface area method. Mol. Inform..

[49] Da Silva A.C., Higgins M.J., Córdoba de Torresi S.I. (2019). The effect of nanoscale surface electrical properties of partially biodegradable PEDOT-co-PDLLA conducting polymers on protein adhesion investigated by atomic force microscopy. Mater. Sci. Eng. C.

