# Supplementary material for: Mechanisms of Polymer–Antigen Binding and Hydrolysis Inhibition: Molecular Dynamics Simulations and Experimental Measurements
Source: Polymers (Basel). 2026 Mar 24;18(7):781. doi: 10.3390/polym18070781 (PMC13074269; doi:10.3390/polym18070781)
Supplement: Supplementary file 1 [file polymers-18-00781-s001.zip › polymers-4206559-supplementary.pdf]

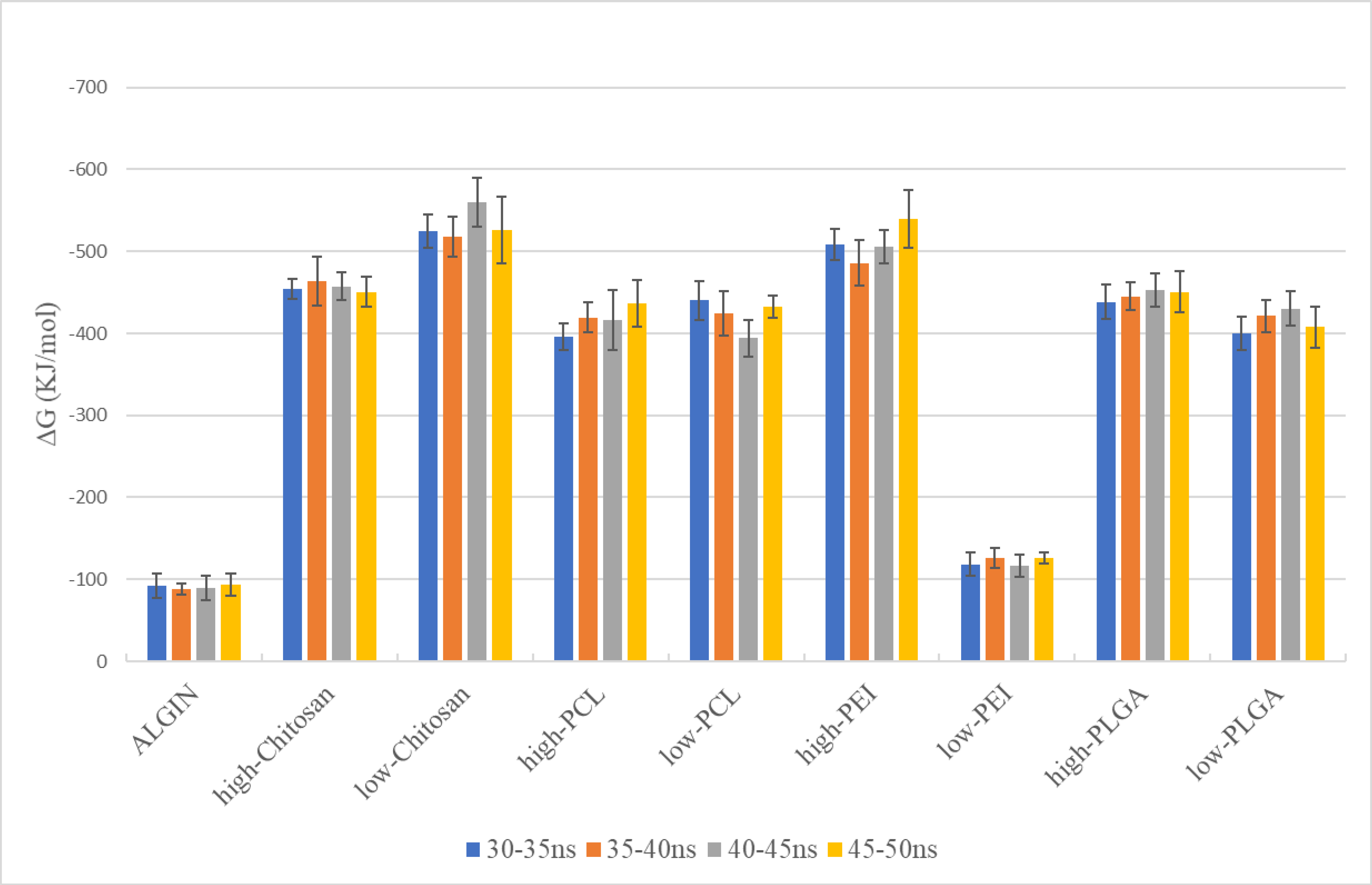

Figure S1. Convergence analysis of polymer–protein binding free energy (ΔG_{bind}) for the last 20 ns of MD simulations.

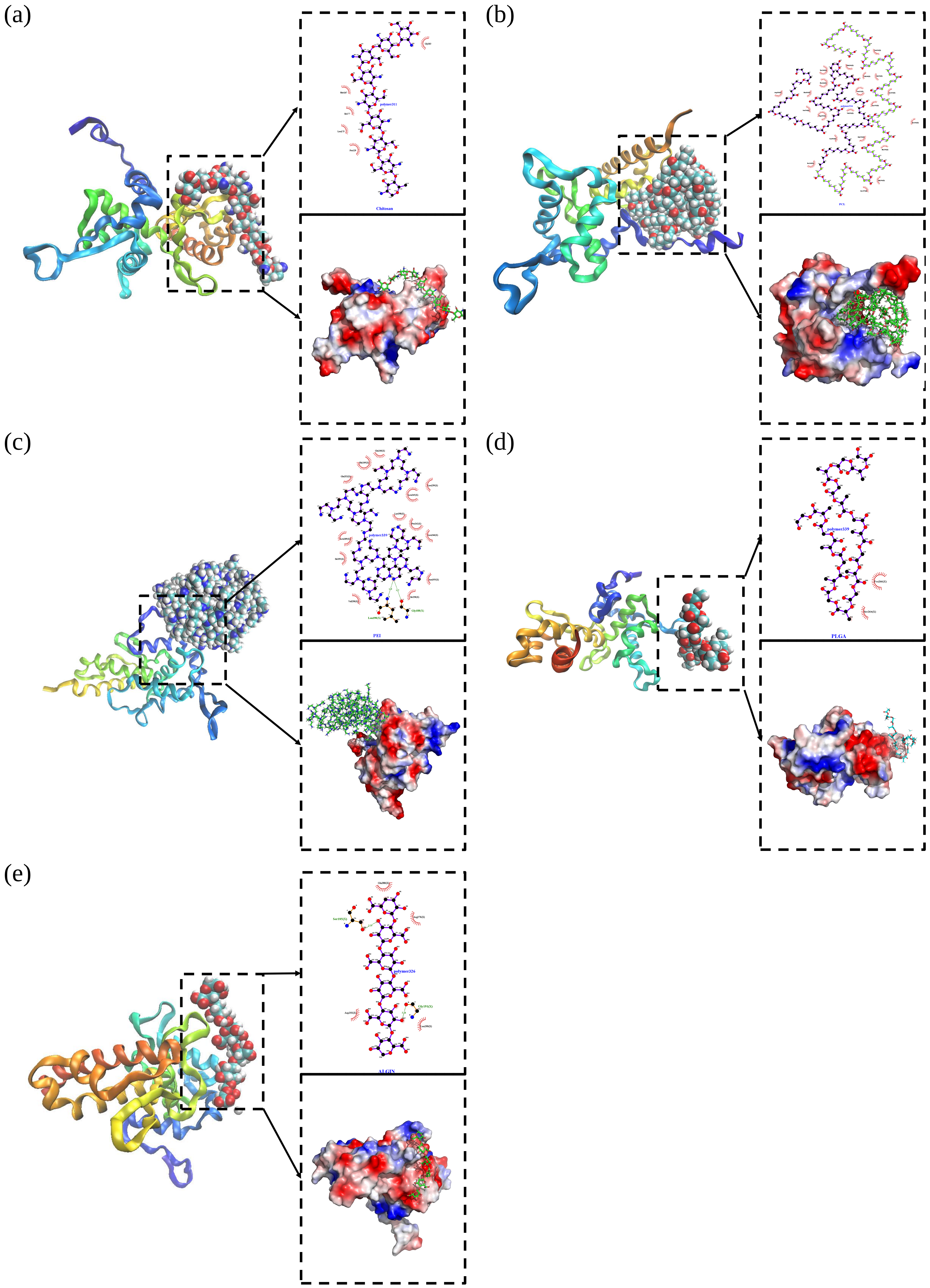

Figure S2. Snapshots, LigPlot diagrams, and electrostatic potential surface distributions showing the binding sites of five types of polymers with the antigen.

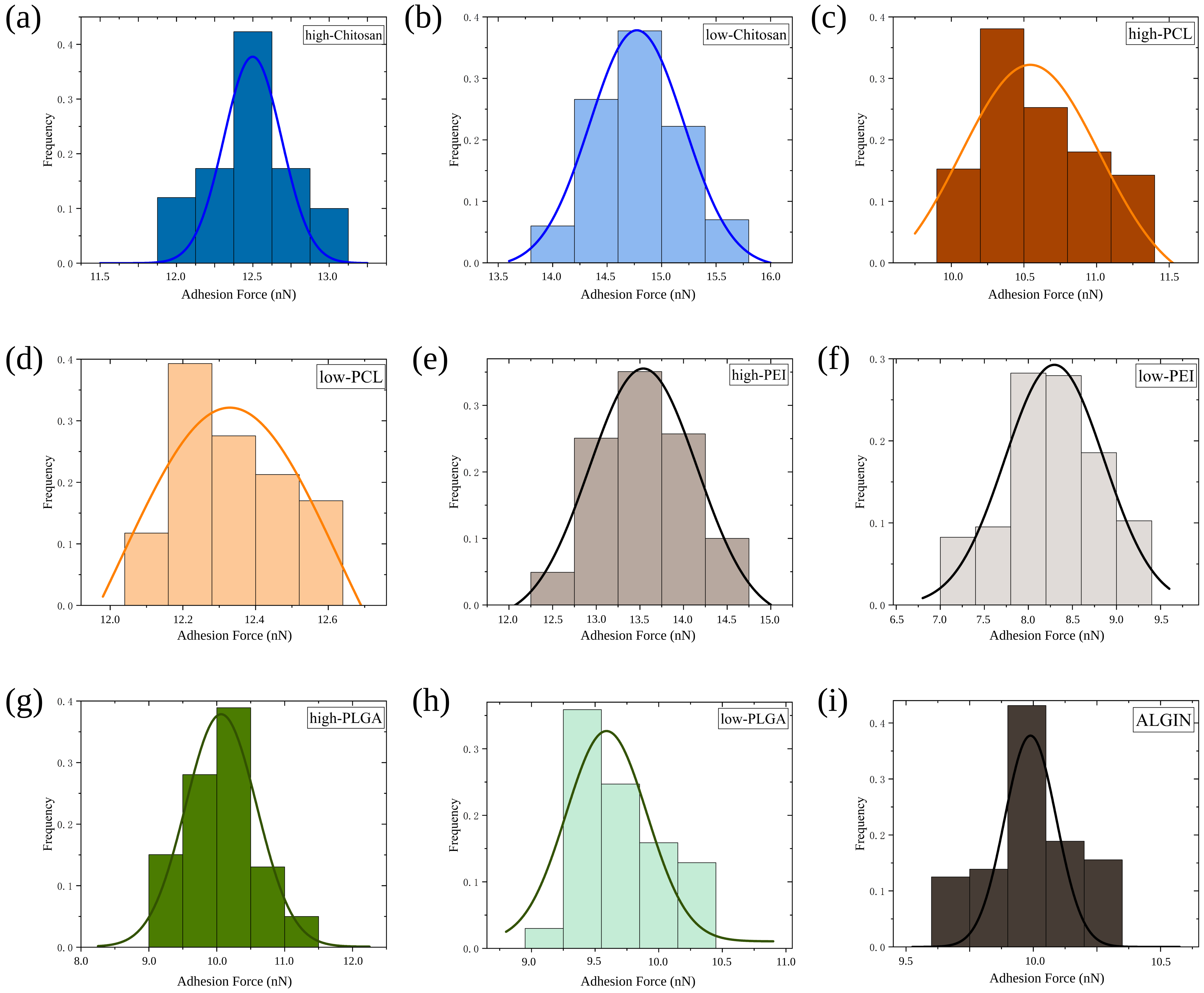

Figure S3. AFM-measured adhesion force distribution curves for nine polymer systems of five types with different degrees of polymerization.

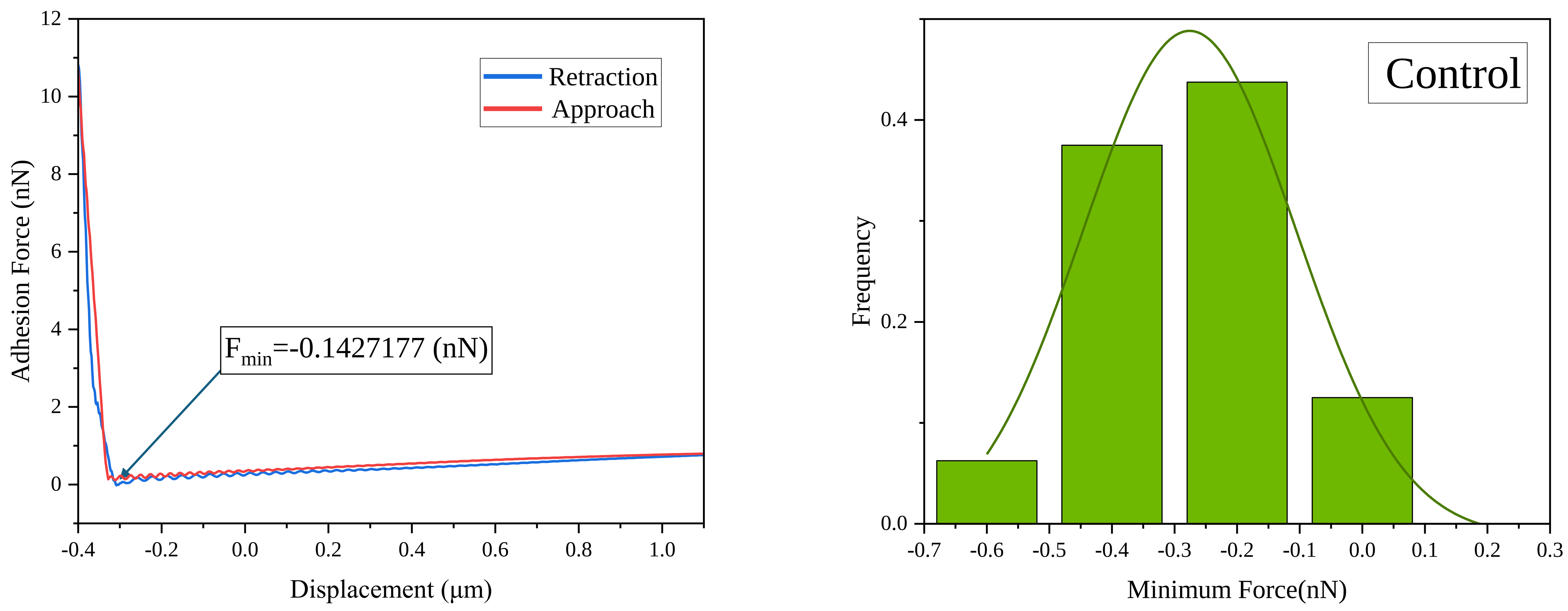

Figure S4. AFM blank control for non-specific adhesion.
Left: Representative force–distance curve from the blank control measurement, showing minimal adhesion events. Right: Distribution of the minimum forces measured across multiple blank curves, with a fitted curve overlaid, representing the baseline non-specific adhesion force. These results establish a reference for differentiating specific polymer–antigen interactions from non-specific adhesion in the AFM experiments.

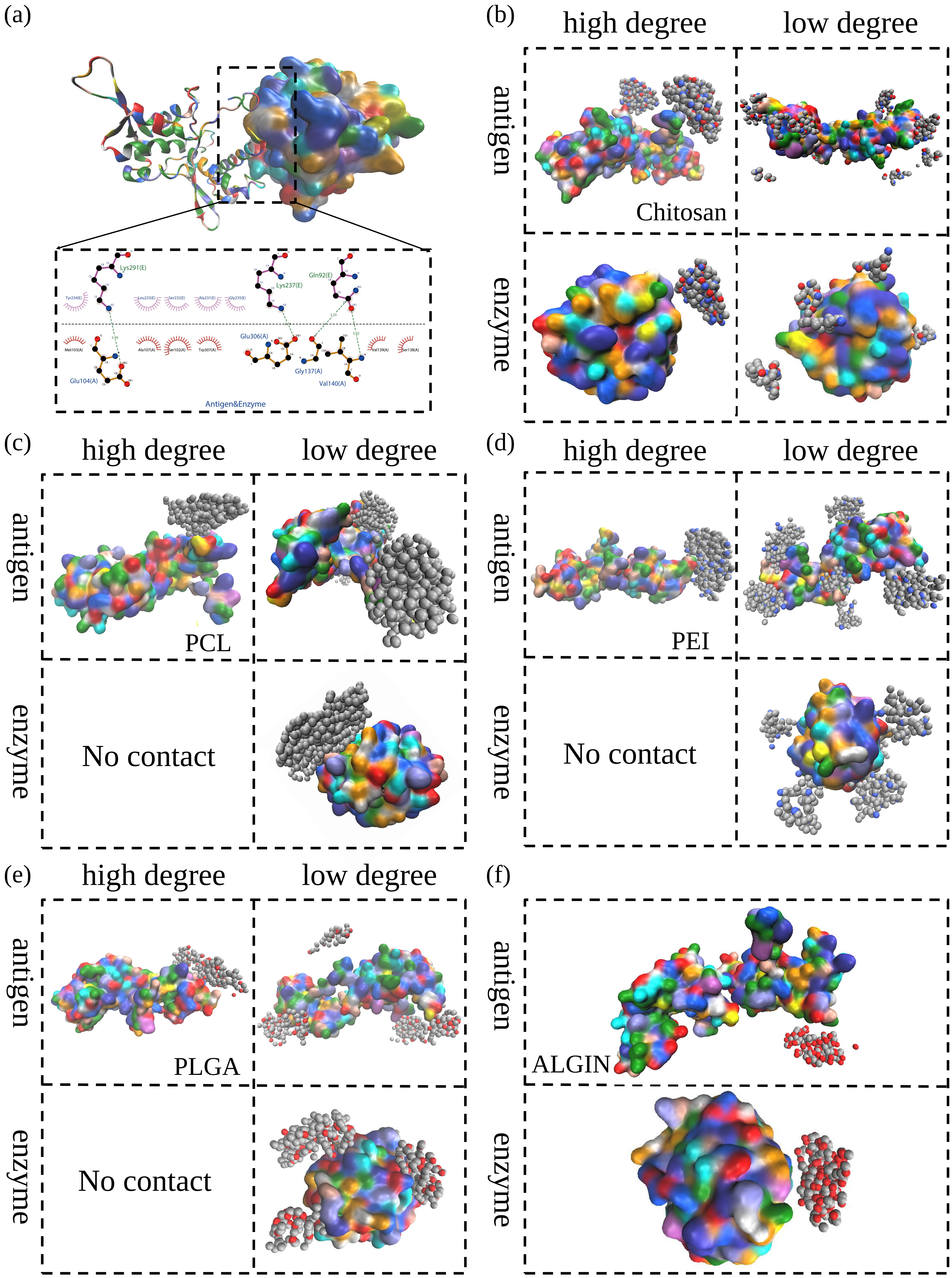

Figure S5. Representative simulation snapshots illustrating the inhibition of antigen–enzyme binding by the nine polymer systems.
